# Le Docteur Magitot

**Published:** 1897-07

**Authors:** 

**Affiliations:** Revue mensuelle de Stomatologie


### Obituary.

### LE DOCTEUR MAGITOT.

The death of Magitot has been a great loss to science and a profound bereavement for stomatology.

At the last moment, the editors of this review, regarding me as the oldest and the most immediate of the pupils of our lamented master, who, in the intimacy of our intercourse, was at times pleased to call me the son of his scientific work, have asked me to express our common sorrow and to recall the part he played as an original scientist, both in France and abroad.

While still suffering from the shock of this cruel bereavement, I should not, at this time and in a few hastily written lines, know how to treat of Magitot's work in detail or according to its merits; but the readers of this journal must not be able to open it without finding in it at least a word of farewell to one who has been its editor from the very first, and who was the veritable founder of stomatology,—our master and our pride.

It is due to his labors, to efforts, indeed, steadily persevered in from the beginning to the close of his career, that he succeeded in making stomatology a special branch in the group of medical sciences and in pointing out its importance and scope; that he gave it a life of its own and created this great scientific movement in regard to a particular point of human investigation, which, before his time, had been almost entirely neglected, and the application of which to the public health had very often been abandoned to empirics and ignorant practitioners.

A convinced and fervent disciple of the doctrines of Auguste Cômte and de Littré, an intimate pupil of our common master, Professor Charles Robin, he had gained from the principles of positive philosophy an ardent love of science, and had found in them an infallible guide for all his researches as well as an unfailing method for all his future labors.

Remaining at the forefront of laboratory work and practice, he pursued at one and the same time the two great methods of experiment and clinical observation, and thus prepared himself for the authoritative work that he was to do later on.

His first researches in anatomy and physiology, which were published for the most part in Robin's journal, and which he was compelled at least partially to revise and modify with our dear master and friend Charles Legros, after microscopy and the methods of investigation had been perfected, secured for him admission to the Society of Biology, where he enjoyed the intimacy of Claude Bernard, Paul Bert, Berthelot, Vulpian, Pouchet, and so many other illustrious men, almost all of whom are now no more. This was also the beginning of those Saturday reunions, where, after the sessions of the Society, literary men like Edmond About, Sainte-Beuve, and Alexandre Dumas joined the scientists, while they were still under the influence of new discoveries, and sought from their discussions, as they were involuntarily but more familiarly continued around the table, those inspirations which for us have given value to their literary masterpieces,—admirable combinations of imagination and truth, in which the original severity of the subject was masked by the graces of form and the subtlety of inventive genius.

With a spirit thoroughly independent, broadly open to new things, and eager for all truths, he devoted himself with passionate interest to that study which of all studies touches us most closely, —the history of man; and he became one of the most active members of the Anthropological Society, whose president he was, after having been for a long time its devoted and indefatigable general secretary, as Broca had been before him, one of the glories of French anthropology, with whom he was also connected by close ties of friendship.

His anthropological researches, which were for him, as he said, a relaxation, were often the occasion for interesting studies of the comparative anatomy of the dental system or of the maxillary formation in prehistoric or later races, and he often turned them to profit in deriving from them ingenious suggestions or even practical considerations of great interest, as, for example, in his treatise on the anomalies of the dental system.

His works on maxillary cysts, on abscesses of the sinus, on tumors of the periosteum, and on phosphorus necrosis soon opened to him the gates of the Surgical Society, and some years later the Medical Academy conferred upon him the enviable and well-merited honor of an admission.

I have only a word to say here on the part which he took in founding the Stomatological Society, of which he has always been the president, and of which he was the soul and the glory to the last moments of his life.

A short time after the organization, still very precarious, of a dental staff in the hospitals, the number of physicians who occupied themselves with diseases of the mouth having slightly increased, and being apparently destined to increase still further, I asked him whether he did not believe that the time had come to unite in one body all those who shared the common bond of similar studies. He approved my project, and replied that, if we desired to found a society under these conditions, he would place his name at our disposal. After this I had no doubts of success. His name was the flag to which all were bound to rally who had any care for scientific interests or for their professional dignity.

In fact, a short time afterwards he assembled at his house those among us whose association was best calculated to establish our effort, and, under his influence, they became the original members of the Stomatological Society.

From that day on he, who had only promised us his name, consecrated to us the best that he had; he devoted to the service of the Society all his zeal for work, all his intellect, and all his strength. Without taking account of distance or fatigue, he more than once sacrificed well-earned and much-needed rest to preside at our sittings, to take part in our discussions, to render them more interesting and luminous, and to share with us the fruits of his long experience.

He struggled with an unconquerable energy against suffering and disease in order to come among us, and we have seen him at times almost overcome with illness, yet unwilling in his great desire to be one with us, to avow himself vanquished. Only for a few months had he been unable to preside over our Society. We all hoped to see him again at our head, when we learned that he had been suddenly stricken down.

Long since an invalid, he had never been willing to abandon the struggle for what he considered the truth. Struck with the dangers to which workmen in the match-factories are exposed, he devoted himself with his customary ardor to the study of the chemical evil, multiplying his published articles in the scientific and political journals, in order to attract to this question of public hygiene the attention of the authorities as well as that of the scientific world, provoking discussions, holding conferences even in other countries, in short, overworking himself with a self-forgetfulness which was bound to terminate, and which did terminate, fatally.

By the powerful and incontestable result of his work, Magitot was the head of a school in the full acceptation of the word,—the head of a school whose profound and lasting influence has made itself felt both abroad and at home. Magitot's work includes not only his publications, treatises of undeniable originality and value, it includes also all the prestige which he gave to stomatology by his communications to learned societies and to the congresses of which he was a member, as well as by the scientific discussions in which he took such an active part. He was the head of a school in knowing how to draw around him pupils to whom he strove to communicate the sacred fire, and who, in their turn, have sought to spread abroad their master's trust, to pass the good word along, and again to make pupils who were at the same time his, and who would once more become instruments for the propagation of his truth.

Moreover, he was a pattern for all, and gave us a great example to consider. By his labor, his profound love of science, and his dignity, he won a universal name and reputation; he attained the highest honors to which a physician can aspire, and secured for himself a place among those men who have made the close of this century illustrious.

Dr. Pietkiewicz,
*Revue mensuelle de Stomatologie.*

